# The impact of endotrophin on the progression of chronic liver disease

**DOI:** 10.1038/s12276-020-00520-8

**Published:** 2020-10-27

**Authors:** Min Kim, Changhu Lee, Dae Yun Seo, Hyojung Lee, Jay D. Horton, Jiyoung Park, Philipp E. Scherer

**Affiliations:** 1 Department of Biological Sciences, School of Life Sciences, Ulsan National Institute of Science and Technology, Ulsan, South Korea; 2 Touchstone Diabetes Center, Department of Internal Medicine, and Department of Cell Biology, University of Texas Southwestern Medical Center, 5323 Harry Hines Boulevard, Dallas, TX USA; 3 Cardiovascular and Metabolic Disease Center, Department of Physiology, College of Medicine, Inje University, Busan, South Korea; 4 Department of Molecular Genetics, and Center for Human Nutrition, University of Texas Southwestern Medical Center, Dallas, TX USA

**Keywords:** Metabolic syndrome, Biological techniques

## Abstract

Non-alcoholic fatty liver disease (NAFLD) is the most common liver disease and can lead to multiple complications, including non-alcoholic steatohepatitis (NASH), cirrhosis, and hepatocellular carcinoma. The fibrotic liver is characterized by the pathological accumulation of extracellular matrix (ECM) proteins. Type VI collagen alpha3 (Col6a3) is a biomarker of hepatic fibrosis, and its cleaved form, endotrophin (ETP), plays a critical role in adipose tissue dysfunction, insulin resistance, and breast cancer development. Here, we studied the effects of the Col6a3-derived peptide ETP on the progression of chronic liver diseases, such as NASH and liver cancer. We used a doxycycline (Dox)-inducible liver-specific ETP-overexpressing mouse model on a NAFLD-prone (liver-specific SREBP1a transgenic) background. For this, we evaluated the consequences of local ETP expression in the liver and its effect on hepatic inflammation, fibrosis, and insulin resistance. Accumulation of ETP in the liver induced hepatic inflammation and the development of fibrosis with associated insulin resistance. Surprisingly, ETP overexpression also led to the emergence of liver cancer within 10 months in the SREBP1a transgenic background. Our data revealed that ETP can act as a “second hit” during the progression of NAFLD and can play an important role in the development of NASH and hepatocellular carcinoma (HCC). These observations firmly link elevated levels of ETP to chronic liver disease.

## Liver disease: Collagen fragment spurs liver inflammation and fibrosis

Localized buildup in the liver of a collagen-derived cleavage fragment plays a critical role in accelerating the development of tissue scarring and liver cancer in mice. Using a transgenic mouse model of fatty liver disease, a team led by Philipp Scherer from the University of Texas Southwestern Medical Center, Dallas, USA, and Jiyoung Park of Ulsan National Institute of Science and Technology, South Korea, showed that liver-specific accumulation of a collagen fragment known as endotrophin leads to activation of inflammatory and fibrotic pathways. The mice subsequently develop metabolic dysfunction, insulin resistance and, with time, cancerous nodules in the liver. The findings suggest that endotrophin accumulation, when it occurs in liver tissue that already contains large depositions of fat, can overwhelm normal liver physiology and accelerate organ damage.

## Introduction

On average, 25% of the population suffers from non-alcoholic fatty liver disease (NAFLD), which is one of the most common liver diseases and is associated with insulin resistance and metabolic syndrome^1^. Early-stage NAFLD usually does not cause any symptoms, but sustained liver damage induced by NAFLD can lead to serious liver disease, including non-alcoholic steatohepatitis (NASH), cirrhosis, and HCC. NASH is characterized by inflammation, immune cell infiltration, and hepatocellular damage in the presence of NAFLD. This disease state is also a known factor that can lead to hepatic fibrosis and liver cancer. HCC was the fourth leading cause of cancer-related deaths from 1980 to 2015 worldwide^2^, and hepatocellular carcinoma (HCC) is the end stage of chronic liver disease, with major risk factors, including viral infections (hepatitis viruses), alcohol intake, NAFLD, and several toxins^3,4^. For the most part, the development of HCC occurs on a background of chronic liver injuries, such as hepatic inflammation and fibrosis in humans^5^.

Collagen type 6 (COL6) is a type of collagen that is made up of three chains (a1, a2, and a3) that are secreted into the extracellular region. Col6 levels are positively associated with the degree of liver fibrosis in NASH^6,7^. Specifically, Col6a3 is associated with metabolic disease and several cancers. Furthermore, COL6A3 expression in adipose tissue was associated with insulin resistance in humans^8^, and human adipocyte-specific COL6A3 knockdown resulted in the development of a unique state of inflammatory resistance via the suppression of MCP1 induction^9^. COL6A3 has also been proposed to be an important marker of colorectal cancer and to serve as a predictive marker of poor prognosis^10^.

Endotrophin (ETP) corresponds to a carboxy-terminally cleaved peptide of COL6A3 that plays a pivotal role in the pathogenesis of metabolic dysfunction and the initiation and progression of various cancers^11,12^. In particular, ETP levels in adipose tissues are increased in obesity, which is accompanied by insulin resistance, and are positively associated with adipose tissue fibrosis and inflammation^12^. Moreover, ETP plays a critical role in cancer settings and can accelerate malignant breast cancer growth^11,13^; accordingly, it is highly expressed in breast cancer and a murine model of chemically induced liver cancer^11,14^. Several reports have shown that the ETP level is an important clinical parameter that correlates with a number of different disease states. Recent studies have shown that increased serum ETP levels are associated with increased mortality in chronic kidney disease^15^, and ETP can be a predictive biomarker for the response to antidiabetic insulin-sensitizer treatments^16^. Inhibition of ETP could improve the therapeutic response to cisplatin-thiazolidinedione combination therapy^17^. Furthermore, ETP promoted diethylnitrosamine (DEN)-induced HCC progression and carbon tetrachloride (CCl_4_)-induced liver injury through JNK activation^14^. However, little is known about its role in the progression of chronic liver diseases accompanied by severe steatosis. In this study, we investigated the roles of ETP in the progression of diseases such as hepatic inflammation, fibrosis and HCC. We generated mice overexpressing ETP exclusively in hepatocytes based on a doxycycline (Dox)-inducible Tet-ON system. Severe NAFLD conditions were achieved by crossing liver-specific ETP-overexpressing mice with liver-specific SREBP1a transgenic mice. We hypothesized that the combination of a heavily steatotic model induced by ectopic overexpression of the transcription factor SREBP1a with the potently profibrotic endotrophin molecule would lead to the progression to HCC in a clinically meaningful way.

## Materials and methods

### Animals

The animal care and experimental protocols were approved by the Institutional Animal Care and Use Committee of the UT Southwestern (UTSW) Medical Center at Dallas, TX, USA and the UNIST, Ulsan, South Korea. Liver-specific albumin (Alb)-Cre and Rosa26-loxP-STOP-loxP-rtTA (Rosa rtTA) mice were obtained from The Jackson Laboratory (Bar Harbor, ME). TRE-ETP mice were generated as described previously^12^. These two lines were backcrossed to the FVB strain for >10 generations. Alb-Cre transgenic mice were bred with Rosa26-loxP-stop-loxP-rtTA mice to obtain animals with liver-specific rtTA expression^14^. These mice were subsequently crossed with TRE-ETP transgenic mice. The resulting triple transgenic mice expressed ETP in the liver only after exposure to Dox in an SREBP1a-transgenic mouse background (expressing human SREBP1a (amino acid 1–460) under the control of the rat *PEPCK* promoter). SREBP1a-Tg (S1a) mice were described previously^18–20^. After genotyping, male mice were randomly assigned to different groups. All experiments were conducted using littermate-controlled mouse cohorts. We fed all groups of mice a Dox-containing normal diet (200 mg/kg of doxycycline, Research Diet, New Brunswick, NJ) at 6 weeks of age until the end of the experiment. The mouse groups used in this experiment were as follows: Alb/Ctrl (Alb-Cre^+/−^;Rosa rtTA^+/−^), Alb/ETP (Alb-Cre^+/−^;Rosa rtTA^+/−^;TRE-ETP^+/−^), S1a/Ctrl (Alb-Cre^+/−^;Rosa rtTA^+/−^;S1a^+/−^), and S1a/ETP (Alb-Cre^+/−^;Rosa rtTA^+/−^;TRE-ETP^+/−^;S1a^+/−^).

### Histological and serological analysis

Tissues were excised and fixed in 10% formalin solution and then stored in 50% ethanol. Embedding, processing, and staining of the tissue samples were performed by the UTSW Molecular Pathology Core or a company (Histoire, Seoul, Korea). For the liver function tests, albumin (ALB), alkaline phosphatase (AlkP), blood urea nitrogen (BUN), bilirubin, aspartate transaminase (AST), and alanine transaminase (ALT) were measured with a VITROS analyzer (Ortho Clinical Diagnostics) in the UTSW metabolic core. Anti-Ki67 (Diagnostic BioSystems, Pleasanton, CA), anti-alpha-SMA (Abcam, Cambridge, UK), anti-F4/80 (Sigma, St. Louis, MO), and anti-myeloperoxidase (Abcam) antibodies were used at a 1:200 dilution for immunohistostaining. The signals were detected with the Lab Vision™ UltraVision™ LP Detection System (Thermo Scientific, Waltham, MA). Apoptosis in paraffin-embedded liver sections was quantitated by terminal deoxynucleotidyl transferase dUTP nick end labeling (TUNEL) Kit (Merck, Burlington, MA) according to the manufacturer’s instructions.

### Lipid measurements

Serum FFA and total cholesterol (Chol) were determined using kits purchased from Wako Diagnostics (Mountain View, CA) and Thermo Fisher (Waltham, MA), respectively. Hepatic triacylglycerol (TG) was measured as previously described^21^.

### Oral glucose and insulin tolerance test

The mice were fasted for 5 h, and blood samples were collected from the tail after the injection of glucose (2.5 g/kg) orally or insulin (0.75 U/kg) intraperitoneally. The blood samples were collected at the indicated time points as previously described^22^.

### ELISA and immunoblotting

ELISA kits for α-fetoprotein (AFP) and TGFβ were purchased from R&D Systems (Minneapolis, MN). Circulating insulin levels were determined using an ELISA kit from APLCO (Salem, NH). A GOLM1 kit was obtained from LSBio (Seattle, WA). The absorbance was measured according to the manufacturers’ manual. Collected liver specimens were homogenized (20 mM Tris (pH 7.4), 5 mM EDTA, 1% NP40, 10 mM Na_4_P_2_O_7_, 100 mM NaF, 2 mM Na_3_VO_4_, 5 μg/mL aprotinin, 5 μg/mg leupeptin, 1 mM phenylmethylsulfonyl fluoride) using a Fast-prep homogenizer (MP Biomedicals, Irvine, CA) and zirconia beads. The protein bands were detected with species-specific secondary antibodies conjugated with infrared dyes, visualized using a Li-Cor Odyssey infrared scanner (Li-Cor Bioscience, Lincoln, NE), and analyzed with Image Studio (Li-Cor Bioscience).

### Real-time qPCR and RNAseq analysis

Total RNA was extracted by a combined method using TRIzol (Invitrogen, Carlsbad, CA) and an RNeasy RNA extraction kit (Qiagen, Hilden, Germany) according to the manufacturers’ instructions. Then, 300 ng of RNA was used as a template for reverse transcription reactions (Invitrogen). The reverse transcription product was appropriately diluted and used for qPCR reactions as previously described^23^. Quantitative real-time PCR was performed with SYBR gene-specific primers on an ABI QuantStudio 5 or 7900 HT sequence detection system (Applied Biosystems, Foster City, CA). Quantification of genes was performed using the 2^−ΔΔCT^ method. Beta-2-microglobulin (*B2m*) was used as an internal control. Primer sets for qPCR are listed in Supplementary Table 1. RNA sequencing (RNAseq) and bioinformatic analyses were carried out by Novogene, Inc. (Sacramento, CA). The prepared libraries were processed with a NovaSeq 6000 machine. Differential expression analysis was performed using the edgeR R package (3.16.5) (RStudio, Boston, MA). Briefly, a *p*-value of 0.005 and a twofold difference in expression were set as the thresholds for identifying significant differential expression. Enrichment analysis of differentially expressed genes was conducted using the ClusterProfiler R package (RStudio). Gene ontology (GO) data with corrected *p*-values < 0.05 were considered significantly enriched by differentially expressed genes.

### Collagen content measurement

The collagen content in the liver was measured by assessing the levels of 4-hydroxyproline using a kit from BioVision (Milpitas, CA) as previously described^24^. In brief, the excised liver tissue (30 mg) was homogenized in distilled water and incubated in 6 N HCl on a heat block for 6 h. Dried supernatants were further incubated with chloramine-T at 25 °C for 10 min. Next, we added DMAB (p-dimethylaminobenzaldehyde) to each well and incubated the sample at 90 °C for 60 min. The absorbance was measured at 560 nm. Other important materials are listed in the key resource table (Supplementary Table 6).

### Statistical analysis

All results are presented as the mean ± standard error. Statistical significance between groups was determined by a two-tailed Student’s *t*-test or one-way ANOVA. *p*-values < 0.05 were considered significant. Statistical analysis and graphs were generated using GraphPad Prism7 software.

## Results

### High-level ETP exposure in the liver for 8 months induces hepatic inflammation, fibrosis, and mild liver damage

**Fig. 1:**
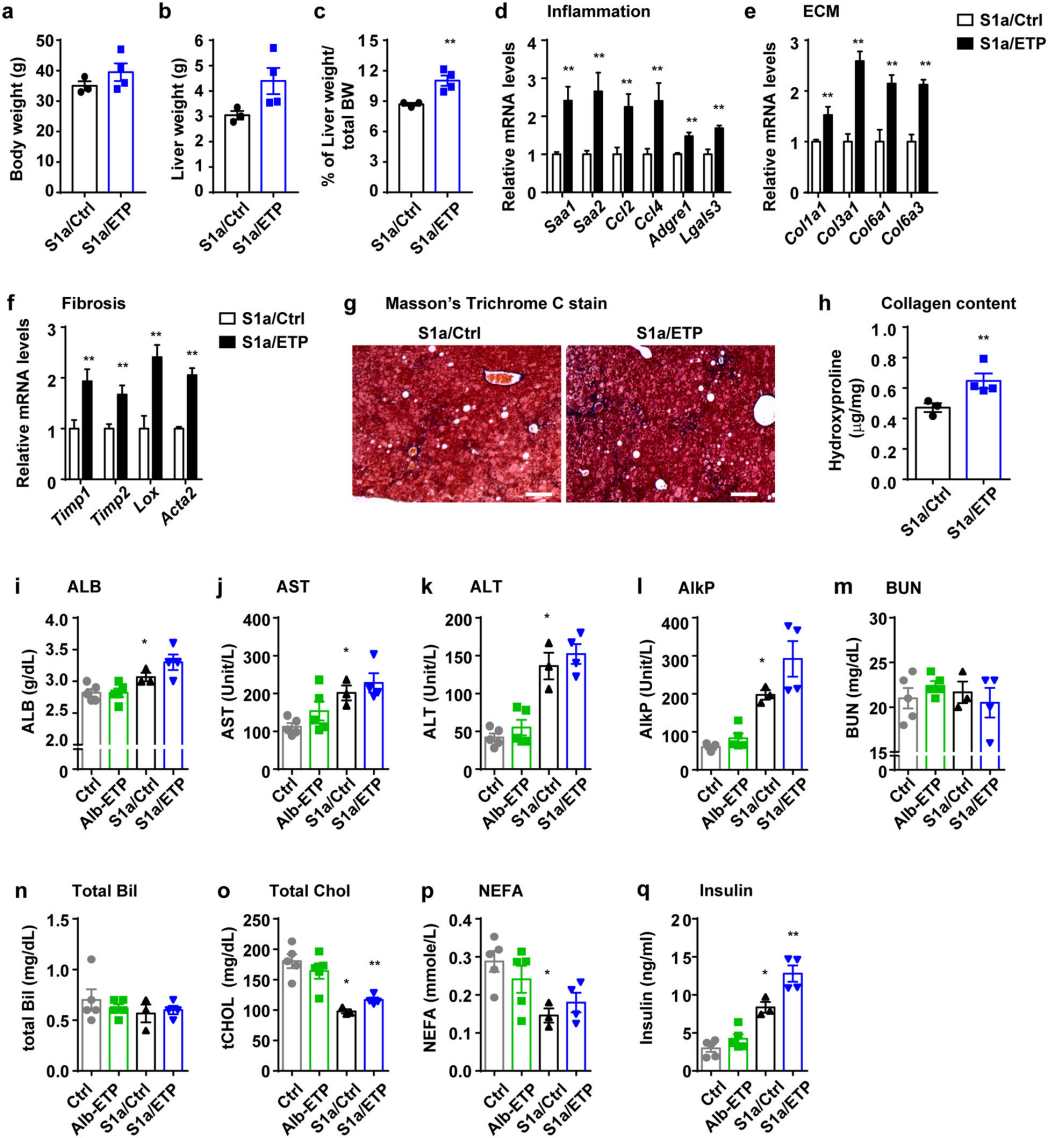
Endotrophin (ETP) exposure for 8 months induces hepatic inflammation and fibrosis and mild liver damage. **a**–**c** Liver and body weights of the mice and percent of liver weight relative to body weight. **d**–**f** Livers were subsequently analyzed for the mRNA expression of collagens, fibrotic markers, and inflammation-associated genes. **g** Masson’s trichrome staining. Bars: 150 µm. **h** Collagen content measurement. **i**–**n** Results of analysis of liver function markers. **p** Serum nonesterified free fatty acid (NEFA) levels. **o** Serum total cholesterol (Chol) levels. **q** Serum fasting insulin levels. *p < 0.05 vs. the livers of the Ctrl mice. **p < 0.05 vs. the livers of the S1a/Ctrl mice.

To determine whether ETP affects hepatic fibrosis and inflammation, we initially generated an inducible, liver-specific ETP transgenic mouse. This model comprises the albumin promoter-driven Cre line, the Rosa26 promoter-driven loxP-stop-loxP-reverse tetracycline-controlled transactivator (rtTA) gene, and a transgenic line carrying tetracycline-responsive elements with mouse ETP (TRE-ETP). To subsequently induce NASH in the presence of ETP, we used steatosis as the starting point for this set of experiments. This condition in the mouse is similar to the progression of human chronic liver disease and is based on our hypothesis that ETP contributes to the progression from simple steatosis to NASH. This triple transgenic mouse was subsequently crossed with a *PEPCK* promoter-driven truncated (constitutively active) SREBP1a (S1a) mouse (Supplementary Fig. 1a). When the mice were fed Dox, ETP gene expression in these quadruple transgenic mice reached ~7-fold that observed in control triple transgenic mouse littermates, and the overexpression was liver-specific, as it was not detected in any other tissues (Supplementary Fig. 1b). ETP expression in combination with the SREBP1a transgene did not lead to any phenotypic changes in the liver when we treated the mice with Dox for 16 weeks. There were no differences in body weight, liver/body weight ratios, or collagen deposition (Supplementary Fig. 2a–c). Lipid accumulation in the liver also did not change. Consistent with these observations, changes in the mRNA levels of marker genes of lipogenesis, fatty acid uptake, fibrosis, and inflammation were not significantly different (Supplementary Fig. 2d–g). Similarly, liver function tests showed no significant differences between the two groups (Supplementary Fig. 2h–k). To test this hypothesis in a more chronic setting, we induced ETP expression for a longer period of time. We treated the transgenic mice with Dox-chow for 8 months. In contrast to the results of the 16-week treatment, the liver weight to body weight ratio was significantly higher in the S1a/ETP mice than the controls (Fig. 1a–c), but we could not find any evidence suggesting that the livers of the S1a/ETP mice are more steatotic than the livers of the controls. Specifically, differences in the mRNA levels of marker genes for lipogenesis and fatty acid uptake were not significantly different between the S1a/Ctrl and S1a/ETP mice (Supplementary Fig. 3a, b). In addition, qPCR showed that the mRNA levels of proinflammatory molecules, collagens, and other markers that represent hepatic fibrosis were increased in the S1a/ETP mice compared to the control mice (Fig. 1d–f). As a result, the accumulation of collagen in the liver was increased (Fig. 1g, h). The levels of liver damage markers, including albumin, BUN, ALT, AST, AlkP, and total bilirubin (Bil), were also measured. The S1a/Ctrl mice showed increased ALT and AlkP levels compared to the control mice but exhibited similar levels of markers to the S1a/ETP mice, with the exception of AlkP (Fig. 1i–n). We subsequently measured total cholesterol and NEFA levels. Consistent with a previous report^25,26^, the levels were decreased in the S1a/Ctrl mice and were not significantly different compared to those in the S1a/ETP animals (Fig. 1o, p). Importantly, fasting insulin levels, as an insulin resistance marker, were increased in the S1a/Ctrl animals, and the levels were higher in the S1a/ETP mice than in the S1a/Ctrl mice (Fig. 1q).

### ETP expression exacerbates inflammation, fibrosis, and liver damage in the SREBP1a transgenic fatty liver mouse model

**Fig. 2:**
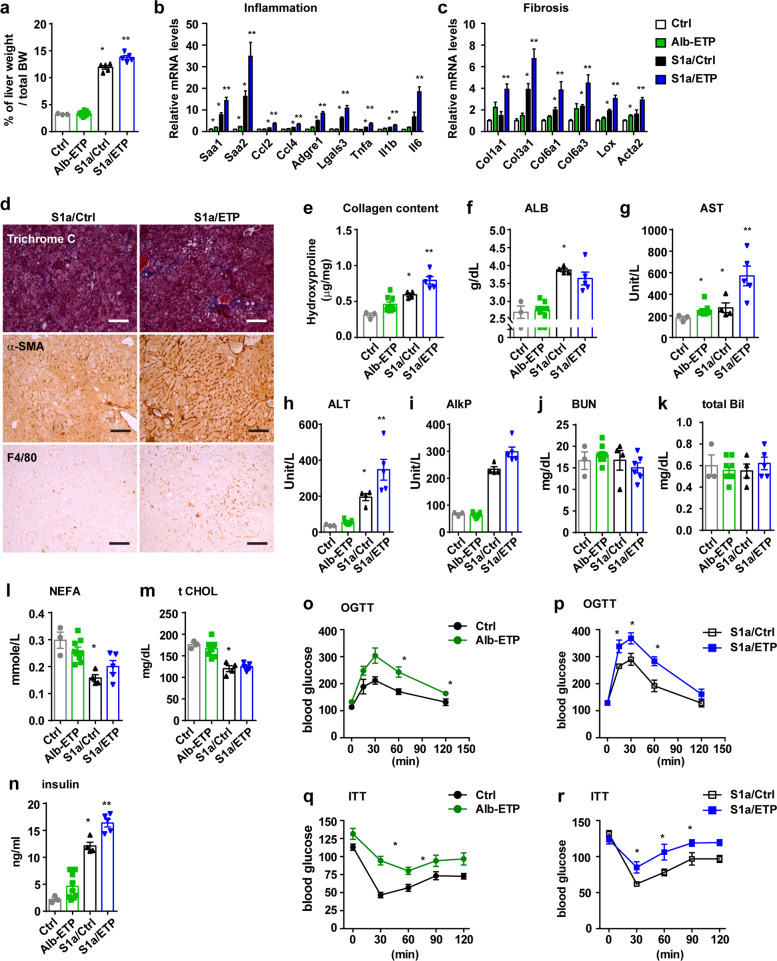
Endotrophin (ETP) exacerbates inflammation, fibrosis, and liver damage in the fatty liver mouse model, S1a transgenic mice and insulin-resistant phenotypes. **a** Mass of the liver normalized to the total body weight of the mice. **b**, **c** qPCR analysis of markers of fibrosis and inflammation in the livers. **d** Masson’s trichrome staining and immunostaining for alpha-SMC and the macrophage marker F4/80 in livers. Bars: 150 µm. **e** 4-Hydroxyproline levels in livers. **f**–**k** Liver function tests in mice. *p < 0.05 vs. the livers of the Ctrl mice. **p < 0.05 vs. the livers of the S1a/Ctrl mice (Ctrl, *n* = 3; Alb-ETP, *n* = 9; S1a/Ctrl, *n* = 4; S1a/ETP, *n* = 5). **l** Serum nonesterified free fatty acid (NEFA) levels. **m** Serum total cholesterol (Chol) level. **n** Serum fasting insulin levels. **o**–**r** Blood glucose levels were measured during an oral glucose tolerance test (OGTT) or insulin tolerance test (ITT) for **o**, **q** Ctrl vs. Alb-ETP and **p**, **r** S1a/Ctrl vs. S1a/ETP. *p < 0.05 vs. the blood glucose level of either the Ctrl or S1a/Ctrl mice. (Ctrl, *n* = 3; Alb-ETP, *n* = 9; S1a/Ctrl, *n* = 4; S1a/ETP, *n* = 5).

As 8 months of ETP expression in the presence of NAFLD induced only mild liver damage (Fig. 1), we extended the study period and fed the mice a Dox diet for 10 months. As expected, the S1a mice exhibited a more severe phenotype, as shown by an increase in hepatic inflammation, the degree of fibrosis, and liver damage markers. Further, the liver weight to body weight ratio was increased (Fig. 2a), and the inflammatory and fibrotic gene expression levels were further enhanced, as expected (Fig. 2b, c). Similar to the results of the Dox 8-month treatment group (Fig. 1c and Supplementary Fig. 3a, b), there was no difference in lipid accumulation between the S1a/Ctrl and S1a/ETP mice (Supplementary Fig. 4d–f). Fibrotic and inflammatory protein analysis showed that the S1a/ETP mice exhibited increased fibrotic (Fig. 2d, e) and inflammatory marker expression in the liver, and the F4/80 protein levels were also elevated (Fig. 2b). Liver damage markers, especially AST and ALT, were significantly elevated in the 10-month treatment group, a significant deterioration compared to that in the 8-month treatment group (Fig. 2f–k).

### Liver-specific ETP mice show an insulin-resistant phenotype

To investigate the metabolic relevance of ETP overexpression in hepatocytes, we analyzed the S1a/ETP mice and the S1a littermate controls after 10 months. We examined whether the expression of ETP with nSREBP1a affects the lipid profiles in the serum. Consistent with previous reports, the levels of TG, NEFA, and total cholesterol in the S1a group were reduced compared to those in the control group^25,26^. Further, serum NEFAs in the S1a/ETP mice were slightly higher than those in the S1a mice alone, but there was no significant difference; however, the cholesterol levels were not different between the two groups (Fig. 2l, m). Notably, basal insulin levels in the S1a/ETP mice were markedly higher than those in the S1a/Ctrl mice. Furthermore, the Alb-ETP mice had elevated levels of fasting blood insulin (Fig. 2n). To further probe for this insulin-resistant phenotype, we performed oral glucose and insulin tolerance tests (OGTT and ITTs). Compared to the control mice, the S1a/ETP mice exhibited significantly higher blood glucose levels throughout the OGTT (Fig. 2o, p), indicating that they were systemically more glucose-intolerant. Furthermore, the S1a/ETP mice had substantially higher blood glucose levels after insulin injection during an ITT (Fig. 2q, r), reflecting impaired insulin sensitivity in these mice.

### Chronic exposure to ETP induces a liver cancer phenotype in SERBP1a transgenic mice

**Fig. 3:**
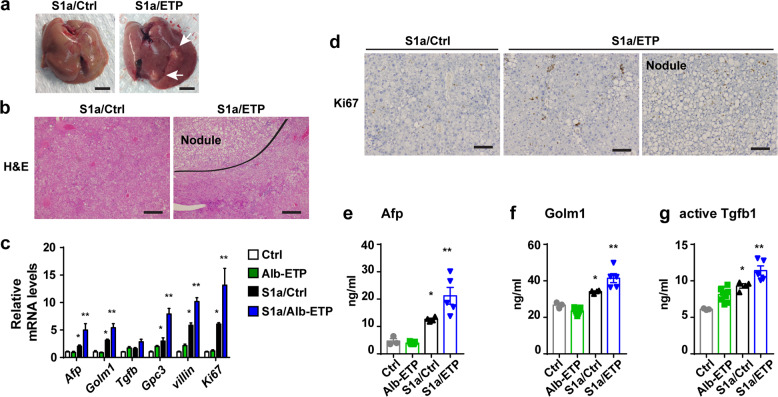
Chronic exposure to endotrophin (ETP) induces liver cancer in the S1a/Ctrl transgenic mice. **a** Representative gross image of the ETP/S1a mouse livers with nodules. Arrowheads indicate nodules and H&E staining of liver tissues; N, nodule. Bars: Gross images (75 mm). H&E staining: (375 µm). **b** Liver cancer markers were determined by qPCR of liver tissues, and the results were normalized based on *B2m* expression. **c** Ki67 staining and **d**–**f** positive liver cancer markers, including serum alpha-fetoprotein, GP73, and active TGF levels, were measured. *p < 0.05 vs. the level of the Ctrl mice. **p < 0.05 vs. the level of the S1a/Ctrl mice. Bars: 150 µm (Ctrl, *n* = 3; Alb-ETP, *n* = 9; S1a/Ctrl, *n* = 4; S1a/ETP, *n* = 5).

Following the 10-month treatment with Dox-chow, surprisingly, we found tumor-like nodules. Specifically, four of five S1a/ETP mice developed nodules, and two of them exhibited very visible and large nodules, whereas none of the control mice (S1a/Ctrl) harbored any visible tumors (Fig. 3a). To characterize these nodules, we performed histological analysis. This analysis revealed that the nodules were filled with lipid droplets/vacuoles, and these structures were quite different from those associated with chemically induced solid liver cancer (Fig. 3b). Specifically, the structure was similar to that of primary clear cell carcinoma of the liver (PCCCL) based on the presence of lipid droplets. To determine the cancerous properties of these lesions, we tested whether the Ki67 levels were altered (Fig. 3d). Importantly, we found significant upregulation of hepatic cancer marker transcripts, including *Afp*, *Golm1*, *Tgfb*, *Gpc3*, *Villin*, and *Ki67* (Fig. 3c). These results were consistent with data obtained upon measuring serum markers. Levels of circulating liver cancer markers such as Afp, Golm1, and active Tgfβ were significantly increased in the S1a/ETP mice compared to the S1a/Ctrl mice (Fig. 3e–g).

### RNASeq revealed significant alteration of neutrophil infiltration in the S1a/ETP mice

**Fig. 4:**
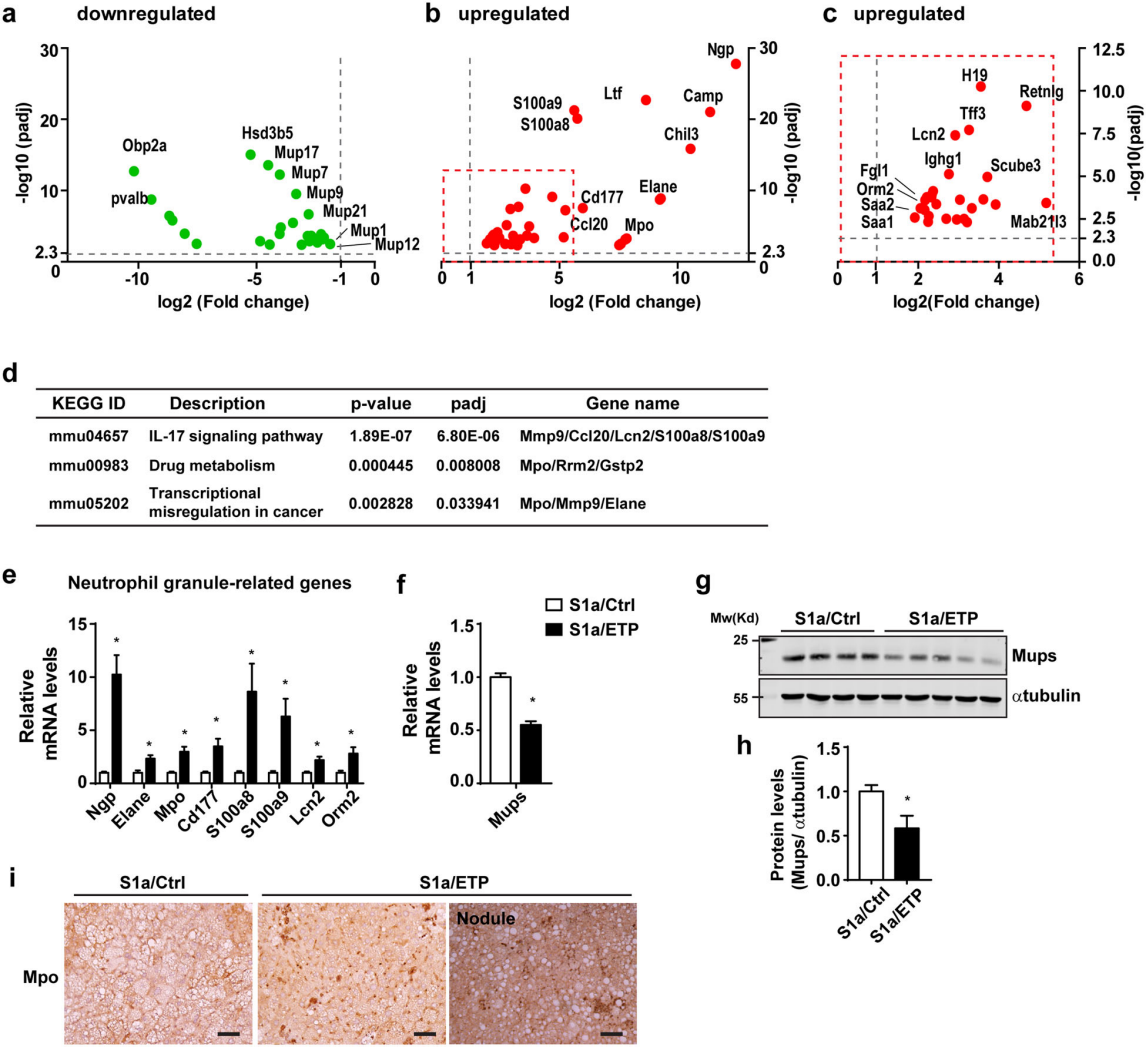
Differential gene expression in the S1a/ETP transgenic mice. **a**–**c** Volcano plot of differentially expressed genes between the two groups. **d** KEGG pathway analysis of these genes. **e**, **f** Confirmation of up- or downregulated genes by qPCR. **g**, **h** Confirmation of the Mup protein levels in the livers of the S1a/Ctrl and S1a/ETP mice. **i** Immunostaining for MPO. Bars: 150 µm. *p < 0.05 vs. the livers of the Ctrl mice. **p < 0.05 vs. the livers of the S1a/Ctrl mice.

To determine the effects of ETP on gene expression and the potential mechanisms through which ETP regulates the progression of chronic liver disease to the development of tumors, we performed an RNAseq analysis of the livers from the S1a/Ctrl and S1a/ETP mice. RNA from three mice was used per group (10-month treatment group). As expected, we observed an increase in the levels of markers that are important for immune responses and inflammation (*Saa1*, *Saa2*, *Lcn-2*, *Ltf*, *and Orosomucoid-2*) and initiating and promoting tumor growth (*Ighg1*, *Scube3*, and *Fgl1*). In addition, genes abundantly expressed in neutrophils (*Elane*, *Mpo*, *Ngp*, *Prtn3*, *Chil3*, *Cd177*, *S100a8*, *S100a9*, and *Lcn-2*) were upregulated (Fig. 4b, c). In contrast, the transcript levels of markers that generally decrease with tumor progression, such as *Mup 1*, *7*, *9*, *7*, and *21* (major urinary proteins), were downregulated in the S1a/ETP mice (Fig. 4a). Interestingly, gene expression levels for the IL17 signaling pathway were elevated in the livers of the S1a/ETP mice (Fig. 4d). Subsequent reactome and gene ontology (GO) analyses showed significant changes mainly in leukocyte degranulation, migration, and other functional components in the immune response (Supplementary Tables 2 and 3). The RNAseq results were then confirmed by qPCR (Fig. 4e, f); consistent with the mRNA levels, the myeloperoxidase (MPO) protein levels were also increased, and the Mup protein levels were decreased in the livers of the S1a/ETP mice (Fig. 4g–i). The top 25 upregulated and downregulated genes in the livers of the S1a/ETP and S1a/Ctrl mice are listed **(**Supplementary Tables 4 and 5**)**. We also confirmed whether the expression of these genes changed in the group treated with Dox for 8 months. The neutrophil markers Ngp, Mpo, and Cd177 showed an increasing trend but no significant change; Elane increased significantly, and the fold change was less than that of the Dox 10-month treatment group **(**Supplementary Fig. 3c). The Mup mRNA and protein levels were decreased in the livers of the S1a/ETP mice compared to the livers of the S1a/Ctrl mice **(**Supplementary Fig. 3d–f). Furthermore, we determined the apoptotic index since changes in the apoptotic program could be an important factor during carcinogenesis. We found that the apoptotic index was not altered between the groups, as evidenced by qPCR analysis of proapoptotic gene expression, TUNEL staining, and Caspase-3 cleavage **(**Supplementary Fig. 4a–c). In summary, our study suggests that ETP accelerates the development and progression of liver disease under steatotic conditions through elevated inflammation and increased neutrophil infiltration.

## Discussion

Numerous studies have shown that the progression of chronic liver disease is not only dependent on genetic and epigenetic factors; ECM deposition is also a major driver of this process. The enhanced production of ECM moieties in various cell types of the liver is strongly associated with hepatic fibrosis, and these changes can eventually lead to HCC. Although the relationship between ECM metabolism and chronic liver disease is well established, the detailed mechanism underlying this process and how changes in ECM can progress to chronic liver disease states, such as liver cancer, remain unclear. A number of different mouse models are currently in use to mimic chronic human liver disease, but models that reflect the progression of such disease states in a clinically relevant manner are very limited.

The lack of useful NASH models has led to the need for spontaneous rodent NASH models for drug discovery and therapeutic studies. Our previous efforts revealed that ETP exacerbates hepatic inflammation and fibrosis upon chemical (CCl_4_ or DEN) treatment or physical treatment (bile duct ligation) in a liver-specific rodent model of ETP expression^14^. Further, to address these unmet needs, we crossed liver-specific ETP mice with liver-specific (*PEPCK* promoter-driven) truncated human SREBP1a (constitutively active) mice. Similar to our previous observations, here, we showed that ETP alone does not promote chronic liver disease progression upon induction for 16 weeks or less under NAFLD conditions.

To further investigate whether ETP can act as a “second hit”, we fed mice Dox for 8 months under steatotic conditions. Indeed, the expression of marker genes related to inflammation and fibrosis was increased in the S1a/ETP mice compared to the S1a/Ctrl mice. After 8 months of exposure to ETP, changes in the S1a/ETP group were mild. Thus, to further exacerbate these changes, we treated mice with ETP for an additional 2 months (10 months in total). As expected, the S1a/ETP mice showed an increased liver weight/body weight ratio, exacerbated inflammation, and a fibrotic phenotype with insulin resistance, as assessed by fasting blood insulin levels and the GTT and ITT results. In this cohort, glucose and insulin intolerance increased in the Alb-ETP mice, suggesting that chronic exposure to ETP without steatosis can contribute to impaired glucose and insulin tolerance. Since our current insights into ETP signaling are limited, we do not fully understand how ETP can induce an insulin-resistant phenotype. This issue should be further clarified in the future. The degree of fat accumulation in the liver can cause various adverse phenotypes; therefore, we measured the hepatic TG levels and lipogenic gene expression in the liver to explore whether the adverse effects were caused by a change in the fat accumulation level. These observations suggest that ETP has no effect on fat accumulation in the liver and acts as an accelerator towards the next stage of chronic liver disease. Nevertheless, we recently demonstrated that ETP can activate the c-Jun amino-terminal kinase (JNK) pathway in the liver^14^ and that this process interferes with insulin action in peripheral tissues^27^; further, we also found that this pathway is activated by inflammatory cytokines. This increased JNK activity and the higher cytokine levels in the livers of the S1a/ETP mice may decrease glucose and insulin tolerance. In addition, the S1a/Ctrl mice are more glucose-intolerant than the control mice, and this phenotype may be caused by lipodystrophy, as shown in previous reports^19,20^. Indeed, the S1a/ETP mice showed severe inflammation and fibrosis, and surprisingly, nodules were observed in the livers of the animals.

The naturally and spontaneously occurring nodules in our mouse model were slightly different from typical liver cancer induced by chemicals, such as CCl_4_ and DEN. However, it was very important to clarify whether these nodules were cancerous. Some studies have shown tumors with similar morphology to that observed in our study. For example, lipid-filled tumors appear in hepatocyte-specific PTEN-deficient mice, SB11 (transposase)-overexpressing mice, and PTEN/p53 inactive mutant mice with HBV transgene expression^28–30^. These tumors develop spontaneously and not via carcinogen injection. However, it was quite clear that in our model, liver cancer marker genes and their plasma levels were elevated (Fig. 3). It is thus necessary to determine which pathways and mechanisms contribute to the dramatic differences in phenotypes between these models. To investigate this, we performed RNAseq analysis, and the results showed that neutrophil markers (*Elane*, *Mpo*, *Ngp*, *Prtn3*, and *Cd177*) and genes mainly and highly expressed by neutrophils (*S100a8*, *S100a9*, *and Lcn2*) were significantly increased. Importantly, inflammatory marker genes (*Saa1*, *Saa2*, *Lcn2*, *and Orm2*) were also notably upregulated. In particular, some cancer markers and proliferative markers were upregulated in the livers of the S1a/ETP mice. Furthermore, increased expression of genes downstream of the IL17 pathway was observed. Neutrophil infiltration in organs is known to have important clinical significance and is particularly critical for both the formation and progression of liver cancer^31^. Importantly, increased neutrophil markers were observed in the Dox 8-month treatment group, indicating that an inflammatory and cancer-prone environment was established around this time point. It is clear that these inflammation-related genes are known to be important for the development of NASH, hepatic fibrosis, and liver cancer^32,33^. Our RNAseq analysis suggested that ETP contributes to tumor formation during severe hepatosteatosis through increased immune cell infiltration, IL17 pathway activation, and inflammation. It has been reported that Saa1 and Saa2 can activate IL17a-dependent neutrophilic inflammation in chronic lung disease^34,35^. Thus, these observations suggest that the infiltration of immune cells, particularly that of neutrophils, accelerates the progression of liver cancer upon ETP expression through an inflammation–IL17–neutrophil axis in chronic steatosis.

Importantly, genes encoding major urinary proteins (MUPs) were also differentially expressed in the livers between the S1a/ETP and S1a/Ctrl mice. MUPs are produced predominantly by the liver. Cancerous livers and even liver tissue with nodules express lower levels of *MUPs*; thus, these molecules were suggested as negative tumor markers for mouse hepatocarcinogenesis^36^. Although the mechanism responsible for such a downregulation in *MUP* RNA in mouse liver cancer remains to be elucidated, the downregulation of *Mup* in hepatic cancer tissue is usually a consequence of neoplastic hepatocyte transformation^36^. We observed that the Mup mRNA and protein levels were increased in the livers of the Dox 8-month treatment group. This finding indicates that the level of Mups can be used as a biomarker for the tumor-prone environment, in addition to being a negative tumor marker. Accordingly, our results suggest that decreased MUP levels in the S1a/ETP mouse livers can be the result of early transformative events leading to hepatic cancer formation.

Collectively, our findings suggest that ETP is an important accelerator that plays a crucial role in the liver and mediates the development of hepatic fibrosis and inflammation. ETP also causes metabolic dysfunction and insulin resistance. Our results further suggest that hepatocyte‐specific ETP-expressing mice in the background of a steatosis-enhancing transgene are a valuable model for the pathogenesis of NASH and for the further progression of NASH to HCC in the presence of steatotic conditions.

## Supplementary information

Supplementary information
