# Supplementary material for: The impact of endotrophin on the progression of chronic liver disease
Source: Exp Mol Med. 2020 Oct 27;52(10):1766–76. doi: 10.1038/s12276-020-00520-8 (PMC8080612; doi:10.1038/s12276-020-00520-8)
Supplement: Supplementary file 1 — Supplementary information [file 12276_2020_520_MOESM1_ESM.docx]

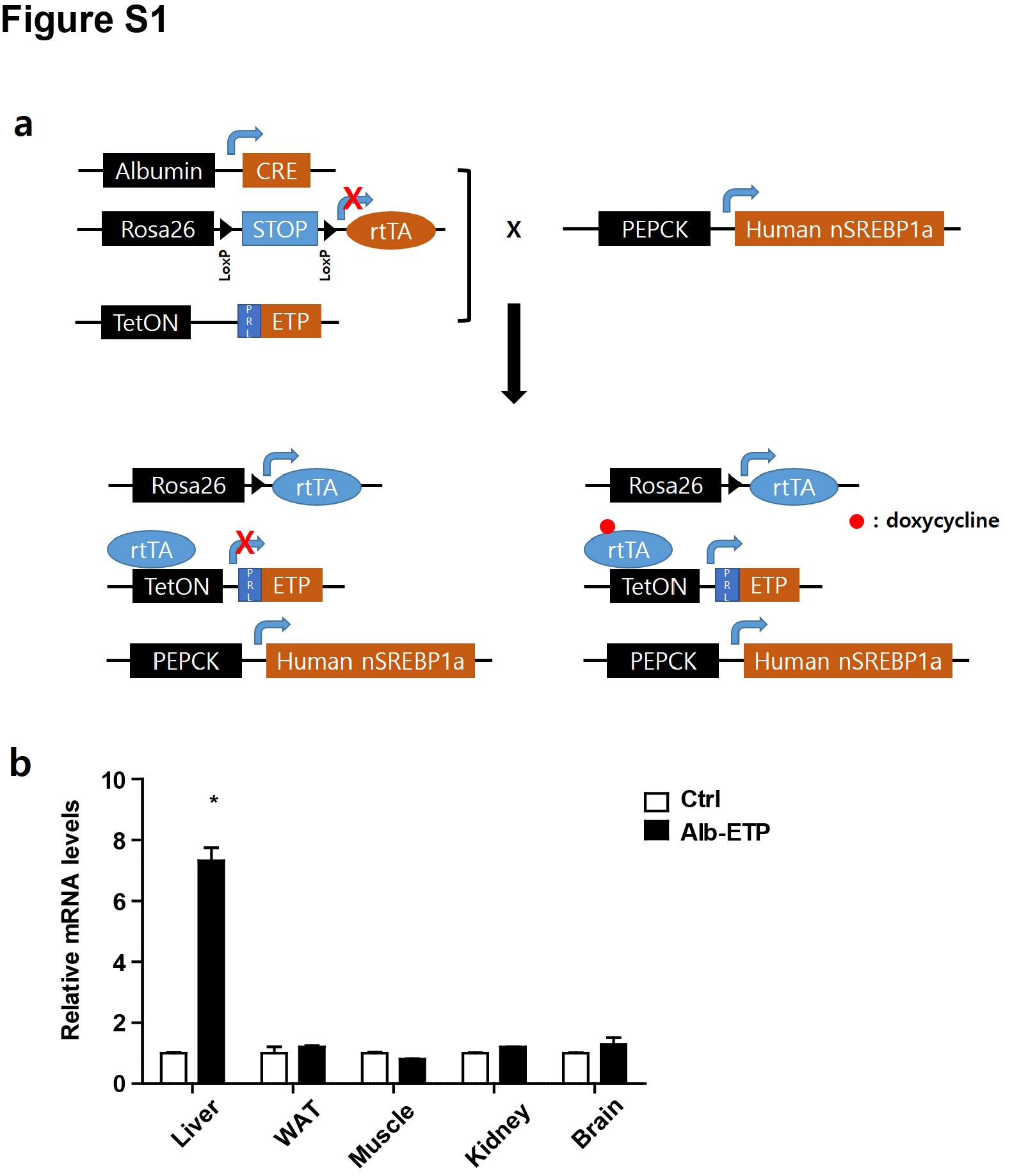


**Supplementary Figure 1. a** Strategy to generate mice with liver-specific inducible endotrophin (ETP) expression in a background of steatosis based on the expression of human nSREBP1a. **b** Validation of mouse model by feeding mice a doxycycline (200 mg/kg) diet for 2 weeks. mRNA expression analysis of *ETP*.


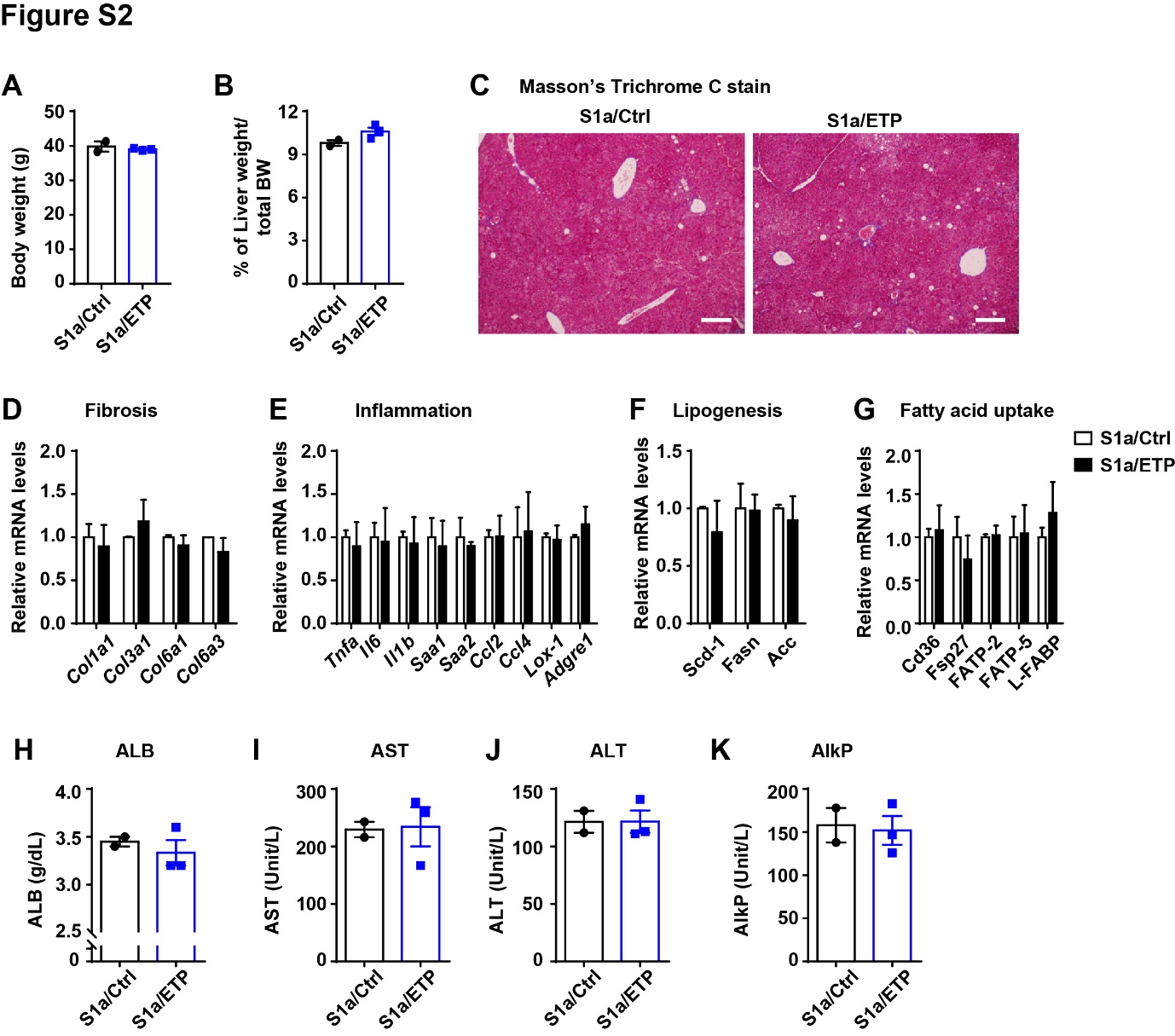


**Supplementary Figure 2.** In the presence of steatosis, the induction of endotrophin (ETP) overexpression for 16 weeks was not sufficient to induce hepatic inflammation and fibrosis. **a** Body weight and **b** liver weight to body weight ratio. **c** Masson’s trichrome staining. **d-g** mRNA analysis of hepatic fibrosis, inflammation, lipogenesis, and fatty acid uptake. **h-k** Liver function tests in mice.

**
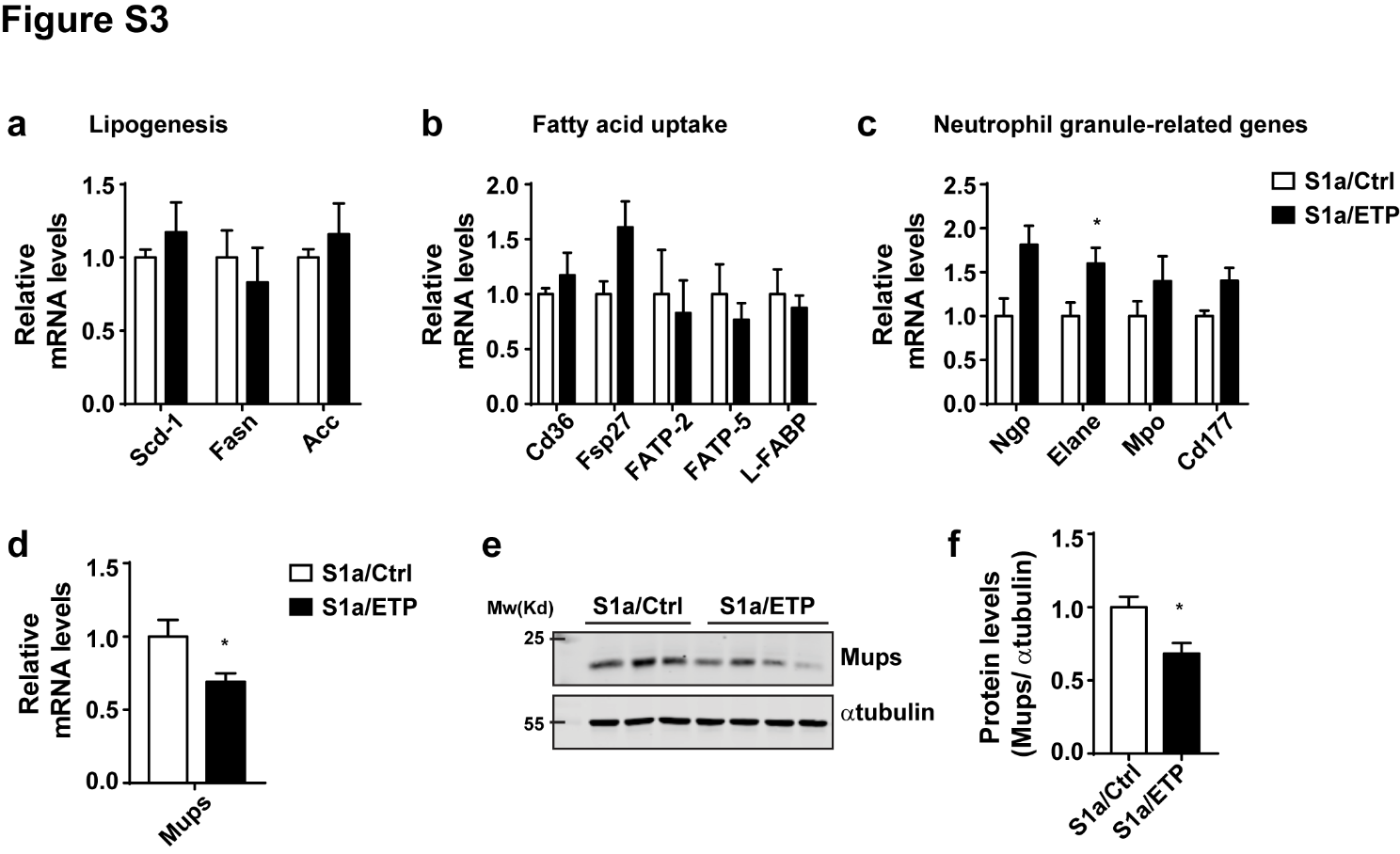
**

**Supplementary Figure 3.** In the presence of steatosis, the induction of endotrophin (ETP) overexpression for 8 months did not change the lipogenic program in the livers of S1a/Ctrl and S1a/ETP mice. **a-c** mRNA levels of hepatic lipogenesis, fatty acid uptake, and neutrophil markers. **d-f** qPCR and Western blot analysis of Mups in the livers of S1a/Ctrl and S1a/ETP mice.

**
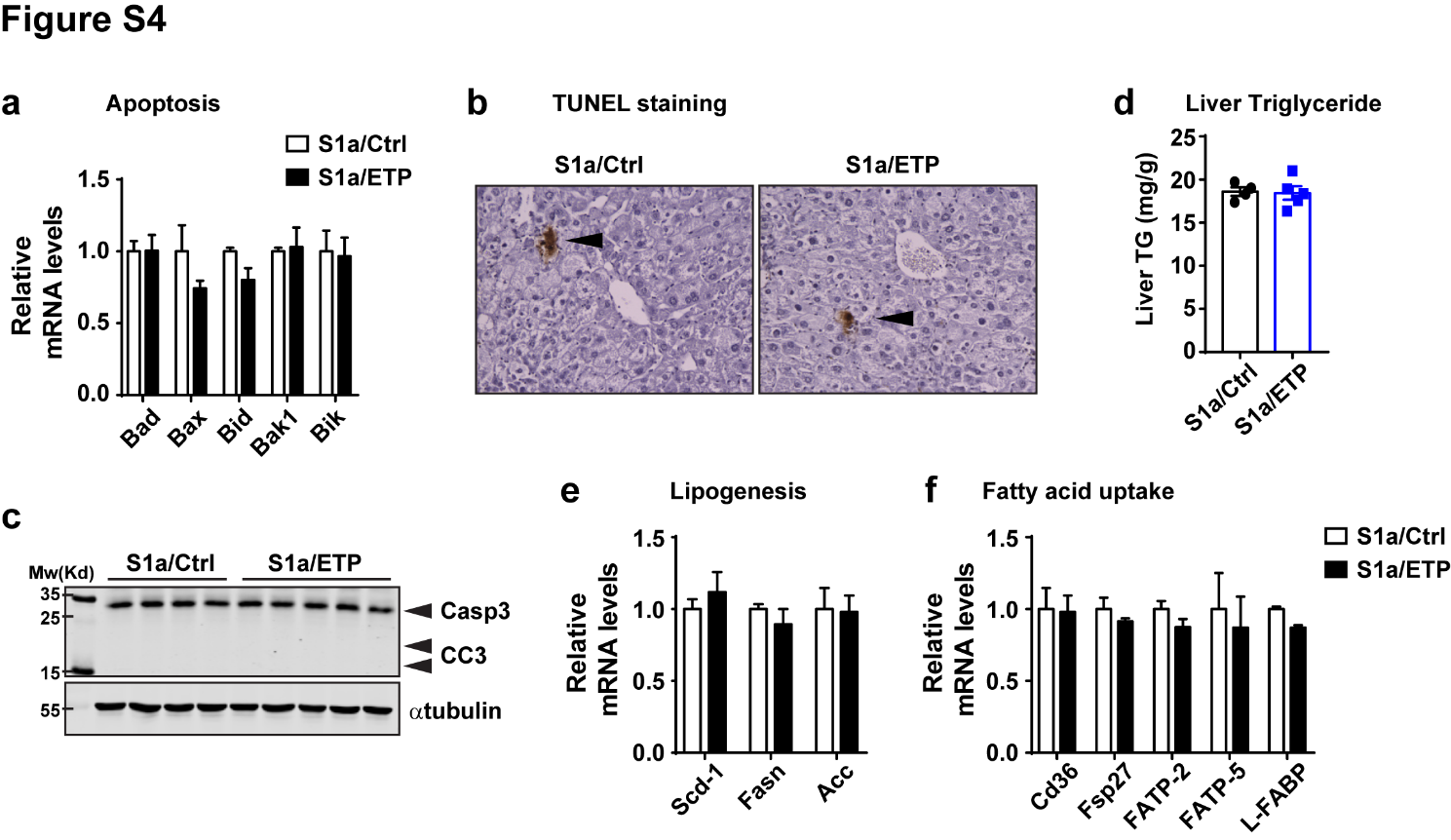
**

**Supplementary Figure 4.** Chronic exposure to endotrophin (ETP) has no effect on hepatic apoptosis and does not change hepatic lipogenesis. **a** mRNA levels of apoptosis markers. **b** TUNEL-staining of the livers. **c** Western blot analysis of Caspase3 (Casp3) and cleaved Casp3 (CC3). **d** Liver TG contents. **e-f** mRNA levels of lipogenesis and fatty acid uptake markers.

**Supplementary Table 1.** Primer sequences

| *Target gene* | Forward (5' to '3) | Reverse (5' to '3) |
| --- | --- | --- |
| *Col1α1* | GTGCTCCTGGTATTGCTGGT | GGCTCCTCGTTTTCCTTCTT |
| *Col3α1* | GGGTTTCCCTGGTCCTAAA | CCTGGTTTCCCATTTTCTCC |
| *Col6α1* | GATGAGGGTGAAGTGGGAGA | CAGCACGAAGAGGATGTCAA |
| *Col6α3* | TCCAGCTTAATCGTGAGGAG | CCATCGATGAGAAAGACCAC |
| *Lox-1* | CCACAGCATGGACGAATTCA | AGCTTGCTTTGTGGCCTTCA |
| *Adgre1* | CTTTGGCTATGGGCTTCCAGTC | GCAAGGAGGACAGAGTTTATCGTG |
| *Tnfα* | GAGAAAGTCAACCTCCTCTCTG | GAAGACTCCTCCCAGGTATATG |
| *IL6* | CCAGAGATACAAAGAAATGATGG | ACTCCAGAAGACCAGAGGAAAT |
| *IL1b* | AAGAAGGTGCTCATGTCCTCATCC | ACTCCAGAAGACCAGAGGAAAT |
| *Saa1* | CATTTGTTCACGAGGCTTTCCAAGGG | TTCCTGAAAGGCCTCTCTTCCATCAC |
| *Saa2* | AGCTGGCTGGAAAGATGGAGACAA | TGTCCTCTGCCGAAGAATTCCTGA |
| *Ccl2* | TTAAAAACCTGGATCGGAACCAA | GCATTAGCTTCAGATTTACGGGT |
| *Ccl4* | GCCCTCTCTCTCCTCTTGCT | GTCTGCCTCTTTTGGTCAGG |
| *Timp1* | GGATTCCGGGAATGACATCTAT | CTGATAACCTGGATGCAGTCGT |
| *Timp2* | CTGATAACCTGGATGCAGTCGT | CCAGCCAGTCCGATTTGA |
| *Lgals3* | TTGAAGCTGACCACTTCAAGGTT | AGGTTCTTCATCCGATGGTTGT |
| *Acta2* | ATCATGCGTCTGGACTTGG | AATAGCCACGCTCAGTCAGG |
| *Afp* | CTTCCCTCATCCCTCCTGCTAC | ACAAACTGGGTAAAGGTGATGG |
| *Golm1* | GCTGGAGAATGTCAACAAGC | CAGTGCCTTCAACTGGTCTT |
| *Gpc3* | CAAGTCACTGCAAGTCACTCG | CGGCCACAGTCCTTACTAAAC |
| *Villin* | TCAAAGGCTCTCTCAACATCAC | AGCAGTCACCATCGAAGAAGC |
| *Ki67* | AGCACAAAGAGACGGTCTAAGA | CTCTGCCTCGTGACTGTGTT |
| *B2m* | TACCACAGGCATTGTGATGG | TTGATGTCACGCACGATTT |
| *Mpo* | CCCACCGAATGACAAGTATC | ACGCCATCTTCATACTCTGC |
| *Cd177* | CGAGGGGTGTTATGAAACAG | CCTGTTCTATGCCTGGTGAG |
| *Elane* | TGTGAACGTATGCACTCTGG | CTCGGATGAAGGAGTCAATG |
| *Lcn2* | ATGGCGAACTGGTTGTAGTC | ACATTTGTTCCAAGCTCCAG |
| *Orm2* | GGGACCCTCTCCAAGTATGT | CCGTTTCTTCTCATCCTTCA |
| *s100a8* | TGAGTGTCCTCAGTTTGTGC | AGATGCCACACCCACTTTTA |
| *s100a9* | ACAAATGGTGGAAGCACAGT | CTCAGCTGATTGTCCTGGTT |
| *Ngp* | CACTCCGCCTTCTAGTCAGA | CCAGGAAGTCGCAGTCTTTA |

**Supplementary Table 2.** Reactome analysis

| Enrichment  Category | ReactomeID | Description | pvalue | padj | geneName |
| --- | --- | --- | --- | --- | --- |
| Reactome | R-MMU-6798695 | Neutrophil degranulation | 1.18E-10 | 8.63E-09 | Mmp8/Mpo/Mmp9/Elane/Lcn2/Fcnb/Prg2/Ltf/Gstp2/  Cd177/S100a8/S100a9/Prtn3/Orm2 |
| Reactome | R-MMU-6803157 | Antimicrobial peptides | 8.74E-10 | 3.19E-08 | Elane/Lcn2/Ltf/S100a8/S100a9/Prtn3 |
| Reactome | R-MMU-1592389 | Activation of Matrix Metalloproteinases | 9.38E-05 | 0.002282 | Mmp8/Mmp9/Elane |
| Reactome | R-MMU-1474228 | Degradation of the extracellular matrix | 0.000287 | 0.005229 | Mmp8/Mmp9/Elane/Scube3 |
| Reactome | R-MMU-8935690 | Digestion | 0.000565 | 0.008245 | Guca2a/Chil3 |
| Reactome | R-MMU-8963743 | Digestion and absorption | 0.001303 | 0.015853 | Guca2a/Chil3 |
| Reactome | R-MMU-5686938 | Regulation of TLR by endogenous ligand | 0.002103 | 0.021933 | S100a8/S100a9 |
| Reactome | R-MMU-140875 | Common Pathway of Fibrin Clot Formation | 0.002571 | 0.023463 | Cd177/Prtn3 |

**Supplementary Table 3.** Gene ontology analysis

| Enrichment  Category | GOID | Description | pvalue | padj | geneName |
| --- | --- | --- | --- | --- | --- |
| GO | GO:0050900 | leukocyte migration | 3.21E-08 | 0.00003 | Mmp9/Elane/Retnlg/Ccl20/Gpsm3/Cd177/  S100a8/S100a9/Prtn3 |
| GO | GO:0060326 | cell chemotaxis | 2.65E-06 | 0.00088 | Retnlg/Ccl20/Gpsm3/S100a8/S100a9/Saa2/Saa1 |
| GO | GO:0061844 | antimicrobial humoral immune response mediated by antimicrobial peptide | 3.03E-06 | 0.00088 | Elane/Ltf/Camp/S100a9 |
| GO | GO:0097529 | myeloid leukocyte migration | 4.03E-06 | 0.00088 | Retnlg/Ccl20/Cd177/S100a8/S100a9/Prtn3 |
| GO | GO:1990266 | neutrophil migration | 4.61E-06 | 0.00088 | Ccl20/Cd177/S100a8/S100a9/Prtn3 |
| GO | GO:0042742 | defense response to bacterium | 8.12E-06 | 0.00096 | Mpo/Elane/Lcn2/Prg2/Ltf/Camp |
| GO | GO:0002523 | leukocyte migration involved in inflammatory response | 1.00E-05 | 0.00096 | Elane/S100a8/S100a9 |
| GO | GO:0097530 | granulocyte migration | 1.19E-05 | 0.00096 | Ccl20/Cd177/S100a8/S100a9/Prtn3 |
| GO | GO:0019730 | antimicrobial humoral response | 1.36E-05 | 0.00097 | Elane/Ltf/Camp/S100a9 |
| GO | GO:0044144 | modulation of growth of symbiont involved in interaction with host | 1.43E-05 | 0.00097 | Mpo/Elane/Camp |
| GO | GO:0044419 | interspecies interaction between organisms | 3.20E-05 | 0.00161 | Mpo/Mmp9/Elane/Fcnb/Ltf/Camp/S100a9 |
| GO | GO:0043901 | negative regulation of multi-organism process | 4.14E-05 | 0.00195 | Mpo/Elane/Fcnb/Ltf/Camp |
| GO | GO:0006959 | humoral immune response | 4.27E-05 | 0.00195 | Elane/Fcnb/Ltf/Camp/S100a9 |
| GO | GO:0019731 | antibacterial humoral response | 4.74E-05 | 0.00206 | Elane/Ltf/Camp |
| GO | GO:0098542 | defense response to other organism | 5.46E-05 | 0.00227 | Mpo/Elane/Lcn2/Prg2/Ltf/Camp/Slfn9 |
| GO | GO:0098869 | cellular oxidant detoxification | 7.09E-05 | 0.00283 | Mpo/Gstp2/S100a8/S100a9 |
| GO | GO:0030595 | leukocyte chemotaxis | 8.92E-05 | 0.00338 | Retnlg/Ccl20/Gpsm3/S100a8/S100a9 |
| GO | GO:0006953 | acute-phase response | 0.00014 | 0.00419 | Saa2/Orm2/Saa1 |
| GO | GO:0031349 | positive regulation of defense response | 0.000157 | 0.00442 | Mmp8/Fcnb/Ltf/Gpsm3/S100a8/S100a9 |
| GO | GO:0002526 | acute inflammatory response | 0.000169 | 0.00461 | Elane/Saa2/Orm2/Saa1 |
| GO | GO:0050729 | positive regulation of inflammatory response | 0.000194 | 0.00487 | Mmp8/Gpsm3/S100a8/S100a9 |
| GO | GO:0002687 | positive regulation of leukocyte migration | 0.000275 | 0.00628 | Mmp9/Elane/Ccl20/Gpsm3 |
| GO | GO:0001819 | positive regulation of cytokine production | 0.000395 | 0.00841 | Mmp8/Elane/Ccl20/Fcnb/Prg2/Gpsm3 |
| GO | GO:0018119 | peptidyl-cysteine S-nitrosylation | 0.000482 | 0.00941 | S100a8/S100a9 |
| GO | GO:0032693 | negative regulation of interleukin-10 production | 0.000482 | 0.00941 | Mmp8/Prg2 |
| GO | GO:0070486 | leukocyte aggregation | 0.000482 | 0.00941 | S100a8/S100a9 |
| GO | GO:0072672 | neutrophil extravasation | 0.000482 | 0.00941 | Cd177/Prtn3 |
| GO | GO:0002227 | innate immune response in mucosa | 0.000561 | 0.01053 | Ltf/Camp |
| GO | GO:0017014 | protein nitrosylation | 0.000561 | 0.01053 | S100a8/S100a9 |
| GO | GO:0034331 | cell junction maintenance | 0.000646 | 0.01190 | Cd177/Prtn3 |
| GO | GO:0051852 | disruption by host of symbiont cells | 0.000738 | 0.01307 | Elane/Camp |
| GO | GO:0051873 | killing by host of symbiont cells | 0.000738 | 0.01307 | Elane/Camp |
| GO | GO:0051851 | modification by host of symbiont morphology or physiology | 0.0008 | 0.01391 | Elane/Ltf/Camp |
| GO | GO:0034612 | response to tumor necrosis factor | 0.001134 | 0.01759 | Ccl20/Lcn2/Ubd/Camp |
| GO | GO:0002237 | response to molecule of bacterial origin | 0.001139 | 0.01759 | Mpo/Elane/Lcn2/Ltf/Camp |
| GO | GO:0030593 | neutrophil chemotaxis | 0.001251 | 0.01901 | Ccl20/S100a8/S100a9 |
| GO | GO:0002573 | myeloid leukocyte differentiation | 0.001381 | 0.02038 | Ltf/Ubd/Gpr55/Prtn3 |
| GO | GO:0071347 | cellular response to interleukin-1 | 0.001384 | 0.02038 | Ccl20/Lcn2/Camp |
| GO | GO:0045638 | negative regulation of myeloid cell differentiation | 0.001478 | 0.02143 | Ltf/Ncapg2/Gpr55 |
| GO | GO:0002251 | organ or tissue specific immune response | 0.001538 | 0.02164 | Ltf/Camp |
| GO | GO:0030099 | myeloid cell differentiation | 0.001748 | 0.02389 | Ltf/Ubd/Ncapg2/Gpr55/Prtn3 |
| GO | GO:0002446 | neutrophil mediated immunity | 0.00212 | 0.02779 | Elane/Cd177 |
| GO | GO:0001818 | negative regulation of cytokine production | 0.002235 | 0.02833 | Mmp8/Elane/Prg2/Ltf |
| GO | GO:0071621 | granulocyte chemotaxis | 0.002246 | 0.02833 | Ccl20/S100a8/S100a9 |
| GO | GO:0030141 | secretory granule | 5.99E-07 | 0.00006 | Mpo/Elane/Tff3/Ngp/Ltf/Camp/Cd177/Prtn3 |
| GO | GO:0035325 | Toll-like receptor binding | 0.000434 | 0.00663 | S100a8/S100a9 |

**Supplementary Table 4.** The top 25 upregulated genes

| Gene_ID | Gene Name | log2FoldChange | pvalue | padj | -LOG10 adj |
| --- | --- | --- | --- | --- | --- |
| ENSMUSG00000032484 | Ngp | 12.46 | 4.63E-33 | 1.63E-28 | 27.787 |
| ENSMUSG00000038357 | Camp | 11.368 | 2.12E-25 | 9.36E-22 | 21.029 |
| ENSMUSG00000040809 | Chil3 | 10.542 | 6.13E-20 | 1.35E-16 | 15.869 |
| ENSMUSG00000020125 | Elane | 9.2825 | 9.72E-13 | 1.27E-09 | 8.896 |
| ENSMUSG00000009350 | Mpo | 9.2395 | 1.52E-12 | 1.85E-09 | 8.733 |
| ENSMUSG00000032496 | Ltf | 8.6531 | 1.61E-27 | 1.90E-23 | 22.722 |
| ENSMUSG00000005800 | Mmp8 | 7.8429 | 1.09E-06 | 0.000557 | 3.254 |
| ENSMUSG00000026835 | Fcnb | 7.7843 | 1.61E-06 | 0.000739 | 3.131 |
| ENSMUSG00000049608 | Gpr55 | 7.7843 | 1.61E-06 | 0.000739 | 3.131 |
| ENSMUSG00000027073 | Prg2 | 7.5925 | 8.28E-06 | 0.003108 | 2.508 |
| ENSMUSG00000092600 | Gm20442 | 7.5225 | 1.27E-05 | 0.004475 | 2.349 |
| ENSMUSG00000052212 | Cd177 | 5.9831 | 2.69E-11 | 2.88E-08 | 7.541 |
| ENSMUSG00000056054 | S100a8 | 5.751 | 1.96E-24 | 7.67E-21 | 20.115 |
| ENSMUSG00000056071 | S100a9 | 5.6249 | 9.80E-26 | 5.50E-22 | 21.260 |
| ENSMUSG00000026166 | Ccl20 | 5.2445 | 5.96E-11 | 5.84E-08 | 7.234 |
| ENSMUSG00000044313 | Mab21l3 | 5.1778 | 6.64E-07 | 0.000366 | 3.437 |
| ENSMUSG00000022651 | Retnlg | 4.6881 | 5.58E-13 | 7.57E-10 | 9.121 |
| ENSMUSG00000018126 | Baiap2l2 | 3.9254 | 8.61E-07 | 0.000447 | 3.350 |
| ENSMUSG00000038677 | Scube3 | 3.7187 | 1.38E-08 | 1.11E-05 | 4.957 |
| ENSMUSG00000023247 | Guca2a | 3.6187 | 3.67E-07 | 0.000223 | 3.651 |
| ENSMUSG00000000031 | H19 | 3.554 | 3.45E-14 | 5.53E-11 | 10.257 |
| ENSMUSG00000017737 | Mmp9 | 3.3326 | 1.56E-06 | 0.000739 | 3.131 |
| ENSMUSG00000024029 | Tff3 | 3.2621 | 1.73E-11 | 1.97E-08 | 7.706 |
| ENSMUSG00000024857 | Cabp2 | 3.2145 | 1.38E-05 | 0.004776 | 2.321 |

**Supplementary Table 5.** The top 25 downregulated genes

| Gene_ID | Gene Name | log2FoldChange | pvalue | padj | -LOG10 adj |
| --- | --- | --- | --- | --- | --- |
| ENSMUSG00000038092 | Hsd3b5 | -5.2513 | 4.43E-19 | 9.18E-16 | 15.04 |
| ENSMUSG00000096688 | Mup17 | -4.5131 | 1.41E-17 | 2.77E-14 | 13.56 |
| ENSMUSG00000062061 | Obp2a | -10.189 | 1.08E-16 | 1.90E-13 | 12.72 |
| ENSMUSG00000073842 | Mup7 | -4.0197 | 3.40E-16 | 5.72E-13 | 12.24 |
| ENSMUSG00000078686 | Mup9 | -3.3264 | 2.12E-13 | 2.99E-10 | 9.52 |
| ENSMUSG00000005716 | Pvalb | -9.4468 | 1.59E-12 | 1.87E-09 | 8.73 |
| ENSMUSG00000038239 | Hrc | -8.6915 | 3.92E-10 | 3.64E-07 | 6.44 |
| ENSMUSG00000066153 | Mup21 | -2.7964 | 2.33E-10 | 2.22E-07 | 6.65 |
| ENSMUSG00000025488 | Cox8b | -8.5617 | 1.68E-09 | 1.52E-06 | 5.82 |
| ENSMUSG00000061462 | Obscn | -3.4609 | 4.06E-09 | 3.49E-06 | 5.46 |
| ENSMUSG00000030785 | Cox6a2 | -3.9758 | 1.97E-08 | 1.54E-05 | 4.81 |
| ENSMUSG00000019787 | Trdn | -8.0449 | 1.67E-07 | 0.000116 | 3.94 |
| ENSMUSG00000030592 | Ryr1 | -4.0261 | 1.79E-07 | 0.000122 | 3.92 |
| ENSMUSG00000042248 | Cyp2c37 | -2.2716 | 2.12E-07 | 0.000141 | 3.85 |
| ENSMUSG00000038576 | Susd4 | -2.6898 | 4.02E-07 | 0.000237 | 3.63 |
| ENSMUSG00000027556 | Car1 | -2.8453 | 4.33E-07 | 0.000246 | 3.61 |
| ENSMUSG00000078683 | Mup1 | -2.6585 | 5.65E-07 | 0.000316 | 3.50 |
| ENSMUSG00000028445 | Enho | -2.6398 | 7.01E-07 | 0.00038 | 3.42 |
| ENSMUSG00000067225 | Cyp2c54 | -2.1462 | 7.70E-07 | 0.000411 | 3.39 |
| ENSMUSG00000040583 | Cyp2b13 | -2.212 | 1.36E-06 | 0.000683 | 3.17 |
| ENSMUSG00000000739 | Sult5a1 | -2.5624 | 1.51E-06 | 0.000739 | 3.13 |
| ENSMUSG00000094793 | Mup12 | -2.5893 | 1.65E-06 | 0.000746 | 3.13 |
| ENSMUSG00000047517 | Dmbt1 | -4.85 | 2.93E-06 | 0.001263 | 2.90 |
| ENSMUSG00000073840 | Mup-ps2 | -2.7656 | 2.94E-06 | 0.001263 | 2.90 |
| ENSMUSG00000022097 | Sftpc | -2.4279 | 4.70E-06 | 0.001973 | 2.70 |

**Supplementary Table 6.** Key Resources Table

| **Reagent or Resource** | **Source** | **Identifier** |
| --- | --- | --- |
| **Antibodies** |  |  |
| alpha SMA | Abcam | Cat# ab5694, RRID:AB_2223021 |
| Myeloperixdase | Abcam | Cat# ab65871, RRID:AB_1139421 |
| Ki-67 (SP6) | Dignostic BioSystems | Cat# RMAB004, RRID:AB_2142363 |
| F4/80 (SP115) | Sigma | Cat# SAB5500103 |
| MUP | Santa Cruz | Cat# Sc-166429, RRID:AB_2017298 |
| Caspase-3 | Cell signaling | Cat# 14220, RRID:AB_2798429 |
| Alpha Tubulin | Sigma | Cat# T5168, RRID:AB_477579 |
| IRDye® 680RD Goat anti-Mouse IgG | LiCor | Cat# 925-68070, RRID AB_2651128 |
| IRDye® 800CW Goat anti-Mouse IgG | LiCor | Cat# 925-32210, RRID AB_2687825 |
| IRDye® 800CW Goat anti-Rabbit IgG | LiCor | Cat# 925-32211,RRID AB_ 2651127 |
|  |  |  |
| **Animals** |  |  |
| Albumin-Cre | The Jackson Laboratory | 003574 |
| Rosa26-LSL-rtTA | The Jackson Laboratory | 005670 |
| TRE-ETP | PMID: 24647224 | N/A |
| PEPCK-SREBP1a | PMID: 8833906 | N/A |
|  |  |  |
| **Commercial Assay** |  |  |
| Mouse Insulin ELISA | ALPCO | Cat# 80-INSMSU-E01 |
| Mouse Golm1 ELISA | LSBio | Cat# LS-F7965 |
| Mouse AFP ELISA | R&D Systems | Cat# MAFP00 |
| Mouse TGF beta ELISA | R&D Systems | Cat# DY1679 |
| Hydroxyproline Colorimetric Assay | BioVision | Cat# K555 |
| NEFA-HR(2) Assay | Wako Diagnostics | Cat# 434-91795 |
| Glycerol Assay | Sigma | Cat# F6342 |
| Total Cholesterol Assay | Thermo Fischer | Cat# TR13421 |
| TUNEL | Merck | Cat# S7100 |
|  |  |  |
| **Software** |  |  |
| Prism7 | GraphPad | https://www.graphpad.com/scientific-software/prism/ |
| Image Studio | LiCor | Ver 5.2.5 |
